# Residential greenness and mortality in oldest-old women and men in China: a longitudinal cohort study

**DOI:** 10.1016/S2542-5196(18)30264-X

**Published:** 2019-01

**Authors:** John S Ji, Anna Zhu, Chen Bai, Chih-Da Wu, Lijing Yan, Shenglan Tang, Yi Zeng, Peter James

**Affiliations:** a Environmental Research Center, Duke Kunshan University, Kunshan, Jiangsu, China; b Global Health Research Center, Duke Kunshan University, Kunshan, Jiangsu, China; c Nicholas School of the Environment, Duke University, Durham, NC, USA; d Department of Social Security, School of Labor and Human Resources, Renmin University of China, Beijing, China; e Department of Geomatics, National Cheng Kung University, Tainan City, Taiwan; f Department of Population Health Science, Duke Medical School, Durham, NC, USA; g Center for the Study of Aging and Human Development, Duke Medical School, Durham, NC, USA; h Center for Healthy Aging and Development Studies, National School of Development, Peking University, Beijing, China; i Department of Population Medicine, Harvard Medical School and Harvard Pilgrim Health Care Institute, Boston, MA, USA

## Abstract

**Background:**

Exposure to natural vegetation, or greenness, might affect health through several pathways, including increased physical activity and social engagement, improved mental health, and reductions in exposure to air pollution, extreme temperatures, and noise. Few studies of the effects of greenness have focused on Asia, and, to the best of our knowledge, no study has assessed the effect on vulnerable oldest-old populations. We assessed the association between residential greenness and mortality in an older cohort in China.

**Methods:**

We used five waves (February, 2000–October, 2014) of the China Longitudinal Healthy Longevity Survey (CLHLS), a prospective cohort representative of the general older population in China. We assessed exposure to greenness through satellite-derived Normalised Difference Vegetation Index (NDVI) values in the 250 m and 1250 m radius around the residential address for each individual included in the study. We calculated contemporaneous NDVI values, cumulative NDVI values, and changes in NDVI from the start of the study over time. The health outcome of the study was all-cause mortality, excluding accidental deaths. Mortality rate ratios were estimated with Cox proportional hazards models, adjusted for age, sex, ethnicity, marital status, geographical region, childhood and adult socioeconomic status, social and leisure activity, smoking status, alcohol consumption, and physical activity.

**Findings:**

Among 23 754 individuals (mean age at baseline 93 years [SD 7·5]) totaling 80 001 person-years, we observed 18 948 deaths during 14 years of follow-up, between June, 2000, and December, 2014. Individuals in the highest quartile of contemporaneous NDVI values had 27% lower mortality than those in the lowest quartile for the 250 m radius (hazard ratio [HR] 0·73, 95% CI 0·70–0·76), and 30% lower mortality for the 1250 m radius (0·70, 0·67–0·74). No clear association was observed for cumulative NDVI measurements and mortality. We did not detect an association between area-level changes in NDVI and mortality.

**Interpretation:**

Our research suggests that proximity to more green space is associated with increased longevity, which has policy implications for the national blueprint of ecological civilisation and preparation for an ageing society in China.

**Funding:**

Bill & Melinda Gates Foundation, US National Institute on Aging, US National Institute of Health, Natural Science Foundation of China, UN Population Fund, China Social Sciences Foundation, and Hong Kong Research Grants Council.

## Introduction

Research suggests that exposure to natural vegetation, or greenness, is linked to improved health outcomes. Greenness is associated with more physical activity, better mental health,^1^, ^2^ maintenance of healthy weight,^3^ higher quality sleep,^4^ better cardiovascular health,^5^ improved cognition,^6^ and faster hospital recovery.^7^ Several mechanisms might explain these observed associations:^8^, ^9^ removal of air pollutants,^10^ provision of accessible space for physical activities,^3^ increased social interaction,^11^ and direct reduction of stress.^11^

Large prospective cohort studies have reported a protective effect of greenness on mortality. Protective associations were seen in the US Nurses Health Study of 108 630 female nurses,^12^ in 1·3 million adults aged older than 25 years in Canada,^13^ and in 575 000 adults aged older than 35 years in Ontario, Canada.^14^ A Swiss national cohort of 4·2 million adults found that residential greenness was associated with reduced risk of mortality independently of other pollution sources.^15^ In urban settings, similar associations were observed in 3144 senior citizens in Tokyo, Japan,^16^ and in 3556 people aged older than 65 years in Hong Kong.^17^ Various systematic reviews have also documented the protective effects of green space on mortality.^18^, ^19^ These published studies on greenness and mortality have been done in regions of the world that have a high human development index, high income per capita, and in populations where people with a higher socioeconomic status live in more desirable areas with more green space. Whether an association exists between greenness and mortality in middle-income countries remains to be seen.

The effect of greenness on the oldest old (those aged >80 years) among the primarily rural population has not been studied in mainland China. With an ageing population and rapid urbanisation in China, this is an important evidence gap. Furthermore, China has diverse geographical and socioeconomic characteristics. We used the Chinese Longitudinal Healthy Longevity Survey (CLHLS), a representative sample of the oldest-old population in China, to examine the association between residential greenness and mortality.

**Research in context**

**Evidence before this study**

We searched PubMed and Google Scholar for studies examining associations between residential greenness and mortality, published in English up to June 1, 2018. We used the combination of search terms “nature”, “natural vegetation”, “greenness”, “green space”, “Normalized Difference Vegetation Index [NDVI]”, and “mortality”. We found eight studies that used NDVI as a form of exposure measurement. Six studies were cohort studies and two were ecological studies; six were done in Europe and North America and two were done in Hong Kong. Five cohort studies reported protective effects of higher levels of NDVI on mortality among adults, including older adults.

**Added value of this study**

In this cohort of 23 754 oldest-old individuals, we observed a lower rate of mortality associated with contemporaneous exposure to greenness, but not with cumulative exposure to greenness. The protective effects of nearby greenness were more pronounced among women and those who exercised. We also found that residential greenness is increasing over time in China. To the best of our knowledge, this is the first study to explore the association between greenness and mortality in the oldest-old population in China, with more than a decade of follow-up.

**Implications of all the available evidence**

China has the largest population of older adults in the world, with increasing numbers of people living longer, alongside an accelerating rate of urbanisation. Our study suggests that green space might affect longevity. This finding provides evidence for policy makers involved in urban planning and health to create healthier cities and better prepare for ageing societies in China.

## Methods

### Study population

The CLHLS covered 22 of 31 provinces in China and was designed to investigate the determinants of healthy longevity among the older Chinese population. This survey only included individuals who were aged 80 years or older between 1998 and 2000, and added individuals aged 65–79 years from 2002. Upon entering the cohort, all individuals were interviewed about determinants of health: socioeconomic characteristics, lifestyle, physical capacity, cognitive function, and psychological status. During each follow-up survey, participants who were still alive were re-interviewed biennially. If an individual had died, their family members were asked about cause of death, health service utilisation, and health status before death. In this analysis, we used data from the 2000, 2002, 2005, 2008, and 2011 waves of the cohort. We assessed survival by use of the follow-up surveys done in 2002, 2005, 2008, 2011, and 2014. Our sample size of five pooled waves from 2000 to 2014 consisted of 38 877 individuals. We excluded individuals if they were lost to follow-up at the first follow-up survey (n=6352), had missing death dates (n=622), lived in regions where greenness could not be calculated (n=341), were younger than 80 years during the study dates (n=7259), or died of accidental causes (n=549). Those who were lost to follow-up in 2002 were more likely to be female, Han Chinese, have physical and cognitive impairments, have few social contacts, and live in urban areas, as compared with those who were included in study follow-up.^20^ People living in areas where Normalised Difference Vegetation Index (NDVI) could not be calculated were more likely to live in coastal regions than were those for whom we could calculate greenness exposure. Our final sample size consisted of 23 754 individuals.

### Assessment of greenness

Exposure to greenness was calculated by use of each individual's residential home address at the time of interview. We assessed greenness through the NDVI, a satellite-image-based vegetation index. This measurement is based on chlorophyll in plants, which absorbs visible light for use in photosynthesis, while leaves reflect near-infrared light. NDVI calculates the ratio of the difference between the near-infrared region and red visible reflectance to the sum of these two measures, ranging from −1·0 to 1·0, with larger values indicating higher levels of vegetative density.^5^, ^12^, ^21^, ^22^ A negative value is often thought of as blue space or water. We did not remove negative NDVI values for our analysis, except when drawing cubic splines for NDVI and mortality.

We obtained NDVI measurements from the Moderate-Resolution Imaging Spectro-Radiometer (MODIS) in the National Aeronautics and Space Administration's Terra Satellite.^23^, ^24^ We linked NDVI imagery to the longitude and latitude of each residential address and calculated greenness in 250 m and 1250 m radii. The 250 m radius is a measure of greenness immediately surrounding the residence. The 1250 m radius was chosen as a measure of greenness within the neighbourhood walking distance of the residence. Walking distance can range from 800 m (0·5 miles) to 1600 m (1 mile); in a previous study, self-reported walking trips were found to have a mean walking distance of 0·7 miles (1126 m).^25^ We added a 500 m buffer analysis to the supplementary analysis.

We assessed NDVI values from February, 2000, to October, 2014, for each of the four seasons. Images from January, April, July, and October were used to represent greenness in winter, spring, summer, and autumn. MODIS has a temporal resolution of 16 days, indicating that two images are available for each month. We used the same day of the year (days 001, 017, 097, 113, 193, 209, 257, 273, 289, and 305) to account for leap years. For each NDVI value, 28 NDVI images were combined to generate a map for the whole mainland.

We calculated three exposure measures: contemporaneous NDVI, to reflect acute exposure to greenness; cumulative average NDVI, to reflect long-term exposure to greenness; and changes in NDVI in the residential area over the course of the follow-up period. Contemporaneous NDVI was the NDVI value at the individual's residential address at the time closest to an event. NDVI values were estimated at date of death for individuals who had died, and at the last interview date for those who were alive and those lost to follow-up. If the duration between these dates was the same, we used NDVI values from the earlier date—for example, if an individual's death was reported on April 15, 2010, and the duration between the death date and both the dates of the extracted seasonal NDVI (April 7, 2010, and April 23, 2010) was the same, we would use the NDVI value at their residential address on April 7, 2010, as their contemporaneous NDVI.

We calculated cumulative NDVI using mean NDVI values at each participant's residential address over the follow-up period.^26^ The follow-up period started from interview dates in 2000 to death dates for deceased individuals, and to the last interview date during the study period for individuals still alive at follow-up and those lost to follow-up. Cumulative average NDVI was the mean of all NDVI values measured over the follow-up period, updated at each time period. The Pearson correlation coefficient between contemporaneous and cumulative NDVI was 0·68.

We defined changes in NDVI as a significant decrease, a non-significant change, or significant increase. We used linear regression to calculate the annual average NDVI from 2000 to 2014 for each participant. If the coefficient of the slope was positive or negative and its p value was less than 0·05, the individual was defined as living in an area with a significant increase or decrease in greenness exposure. If the p value was larger than 0·05, the individual was defined as living in an area with no significant change in greenness exposure.

### Assessment of mortality

We used all-cause mortality as our health outcome, with deaths that occurred between 2000 and 2014 reported by the next of kin. We excluded the 549 (2·3%) individuals whose deaths resulted from accidental causes from the analysis. We were able to obtain cause-specific mortality information for 2476 (13·1%) of 18 948 deaths.^20^

### Covariates

During each interview the assessors measured a range of demographic, behavioural, and socioeconomic covariates ascertained from the baseline survey in 2000, 2002, 2005, 2008, and 2011, including age, sex, ethnicity, marital status, geographical region of residence, childhood socioeconomic status, adult socioeconomic status, social and leisure activity, smoking status, alcohol consumption, and physical activity. Interviewees were encouraged to answer as many questions as possible. If they were unable to answer questions, a close family member or another proxy, such as a caregiver, provided answers.^20^

Age was calculated according to self-reported birth date, based on Chinese lunar calendar dates, and converted to Georgian calendar dates. Age was calculated by subtracting the interview date from the converted birth date. Reported date of birth was verified by family members, genealogical records, ID cards, and household registration booklets. If any individual was reported to be older than 105 years, additional evidence was obtained from local government committees.

Ethnicity was divided into two categories: Han Chinese or ethnic minority (Hui, Korean, Manchurian, Mongolian, Yao, Zhuang, and others). We generated a binary variable to assess marital status: married and living with spouse or not married at interview (separated, divorced, widowed, or never married). We considered geographical region on the basis of residential address to account for climate and dietary differences: central China (Henan, Hubei, and Hunan provinces), eastern China (Anhui, Fujian, Jiangxi, Jiangsu, Shandong, Shanghai, and Zhejiang provinces), northeastern China (Heilongjiang, Jilin, and Liaoning provinces), northern China (Hebei, Shanxi and Tianjin provinces), northwestern China (Shaanxi province), southern China (Guangdong, Guangxi, and Hainan provinces), and southwestern China (Chongqing and Sichuan provinces). Smoking status was dichotomised as “smoker at present” or “non-smoker at present”. A similar approach was taken to define the status of alcohol consumption and physical activity: “drinker at present” or “non-drinker at present” and “exercise (walking, running and ball games) at present” or “no exercise at present”.

We scored questions pertaining to childhood socioeconomic status, adult socioeconomic status, and social and leisure activities with 0 (lower socioeconomic status) or 1 (higher socioeconomic status) to define scales for these measures. Childhood socioeconomic status was assessed by five questions (adequate medication for childhood illnesses, whether the respondent frequently went to bed hungry in childhood, whether both parents were alive at age 10 years, father's occupation [white collar *vs* other], and place of birth [rural *vs* urban]), leading to a total ranging from 0 to 5.^27^ Adult socioeconomic status was assessed by four questions (current residence [urban *vs* rural], education level [having more than 1 year of education *vs* not], economic independence [having retirement earnings *vs* no income], and primary lifetime occupation [white collar *vs* other]), leading to a total ranging from 0 to 4.^27^, ^28^ Social and leisure activity was assessed by seven questions (whether a respondent did gardening, personal outdoor activities excluding those specifically done for exercise, raised poultry or pets, reading, playing cards or mah-jong, listening to the radio or watching TV, and participating in organised social activities), leading to a total ranging from 0 to 7. ^28^

### Statistical analysis

We estimated mortality rate ratios using Cox proportional hazard models, with survival time measured in months from the first interview date to the recorded death date or last interview date up to 2014. We calculated hazard ratios (HRs) and 95% CIs to indicate associations between greenness exposure measured by NDVI and mortality. We adjusted for covariates that could be potential confounders or predictors of mortality: age, sex, ethnicity, marital status, geographic region, childhood socioeconomic status, adult socioeconomic status, social and leisure activity, smoking status, alcohol consumption, and physical activity. We reported results of Cox proportional hazard models for contemporaneous NDVI, cumulative NDVI, and changes in NDVI, in the 250 m and 1250 m radius. We analysed for effect modification by multiplying per-quartile NDVI by potential modifier variables, then did a stratified analysis by these variables. We used three knots cubic splines to explore non-linearity.^29^, ^30^ We used Stata, version 14.0, for survival analyses and splines.

### Role of the funding source

The funders of this study had no role in study design, data collection, data analysis, data interpretation, or writing of the report. The corresponding author had full access to all the data in the study and had full responsibility for the decision to submit for publication.

## Results

Among 23 754 individuals, the mean age was 93 years (SD 7·5) at baseline. 9041 (38·1%) participants were men and 18 879 (79·5%) lived in rural regions (table 1). The median contemporaneous NDVI for the 250 m radius was 0·38 (25th to 75th percentile 0·22–0·57), and the cumulative average NDVI was 0·44 (0·30–0·52). Individuals living in an area with higher levels of greenness exposure were more likely to be younger, male, of ethnic minority, have a lower childhood and adult socioeconomic status, have higher levels of social and leisure activity, and to drink alcohol than were those living in an area with lower levels of greenness exposure.

**Table 1:** Characteristics of CLHLS participants by quartile of cumulative average NDVI within 250 m buffer, from 2000–14 (n=23 754)

		**Total (n=23 754)**	**Greenness quartile 1 (n=5961)**	**Greenness quartile 2 (n=5923)**	**Greenness quartile 3 (n=5928)**	**Greenness quartile 4 (n=5942)**	**p value**
Contemporaneous NDVI		0·38 (0·22–0·57)	0·13 (0·07–0·18)	0·29 (0·25–0·33)	0·47 (0·42–0·52)	0·68 (0·62–0·75)	..
Cumulative average NDVI		0·44 (0·30–0·52)	0·19 (0·15–0·24)	0·39 (0·35–0·42)	0·48 (0·46–0·50)	0·57 (0·54–0·61)	..
Age, years		92·89 (7·46)	92·71 (7·46)	92·46 (7·44)	92·93 (7·52)	93·47 (7·41)	<0·0001
Age group, years							
	80–99	8274 (34·8%)	2113 (35·4%)	2207 (37·3%)	2084 (35·2%)	1870 (31·5%)	<0·0001
	90–99	8211 (34·6%)	2026 (34·0%)	2021 (34·1%)	2038 (34·4%)	2126 (35·8%)	..
	≥100	7269 (30·6%)	1822 (30·6%)	1695 (28·6%)	1806 (30·5%)	1946 (32·7%)	..
Sex							
	Men	9041 (38·1%)	2406 (40·4%)	2267 (38·3%)	2167 (36·6%)	2201 (37·0%)	0·0001
	Women	14 713 (61·9%)	3555 (59·6%)	3656 (61·7%)	3761 (63·4%)	3741 (63·0%)	..
Ethnicity							
	Han Chinese	22 287 (93·8%)	5751 (96·5%)	5691 (96·1%)	5534 (93·4%)	5311 (89·4%)	<0·0001
	Ethnic minority	1467 (6·2%)	210 (3·5%)	232 (3·9%)	394 (6·6%)	631 (10·6%)	..
Marital status							
	Currently married and living with spouse	3927 (16·5%)	1116 (18·7%)	1023 (17·3%)	912 (15·4%)	876 (14·7%)	<0·0001
	Not married	19 827 (83·5%)	4845 (81·3%)	4900 (82·7%)	5016 (84·6%)	5066 (85·3%)	..
	Childhood socioeconomic status	1·42 (1·07)	1·73 (1·19)	1·36 (1·04)	1·28 (0·99)	1·30 (0·98)	<0·0001
	Adult socioeconomic status	0·69 (0·98)	1·37 (1·20)	0·58 (0·87)	0·41 (0·72)	0·38 (0·69)	<0·0001
	Social and leisure activity index	1·74 (1·42)	1·96 (1·49)	1·77 (1·40)	1·65 (1·38)	1·60 (1·38)	<0·0001
Smoking status							
	Yes	3602 (15·2%)	795 (13·3%)	1022 (17·3%)	899 (15·2%)	886 (14·9%)	<0·0001
	No	20 152 (84·8%)	5166 (86·7%)	4901 (82·7%)	5029 (84·8%)	5056 (85·1%)	..
Drinking status							
	Yes	4444 (18·7%)	944 (15·8%)	1114 (18·8%)	1208 (20·4%)	1178 (19·8%)	<0·0001
	No	19 310 (81·3%)	5017 (84·2%)	4809 (81·2%)	4720 (79·6%)	4764 (80·2%)	..
Physical activity							
	Yes	6002 (25·3%)	2006 (33·7%)	1553 (26·2%)	1317 (22·2%)	1126 (18·9%)	<0·0001
	No	17 752 (74·7%)	3955 (66·3%)	4370 (73·8%)	4611 (77·8%)	4816 (81·05%)	..
Geographical region							
	Central China	3651 (15·4%)	600 (10·1%)	783 (13·2%)	1203 (20·3%)	1065 (17·9%)	<0·0001
	Eastern China	9463 (39·8%)	2332 (39·1%)	2307 (38·9%)	2334 (39·4%)	2490 (41·9%)	..
	Northeastern China	1571 (6·6%)	794 (13·3%)	612 (10·3%)	110 (1·9%)	55 (0·9%)	..
	Northern China	1104 (4·6%)	709 (11·9%)	271 (4·6%)	103 (1·7%)	21 (0·4%)	..
	Northwestern China	266 (1·1%)	84 (1·4%)	74 (1·2%)	72 (1·2%)	36 (0·6%)	..
	Southern China	4533 (19·1%)	663 (11·1%)	890 (15·0%)	1141 (19·2%)	1839 (30·9%)	..
	Southwestern China	3166 (13·3%)	779 (13·1%)	986 (16·6%)	965 (16·3%)	436 (7·3%)	..

Data are n (%), mean (SD), or median (25th to 75th percentile). NDVI=Normalised Difference Vegetation Index. CLHLS=China Longitudinal Healthy Longevity Survey.

**Figure 1:**
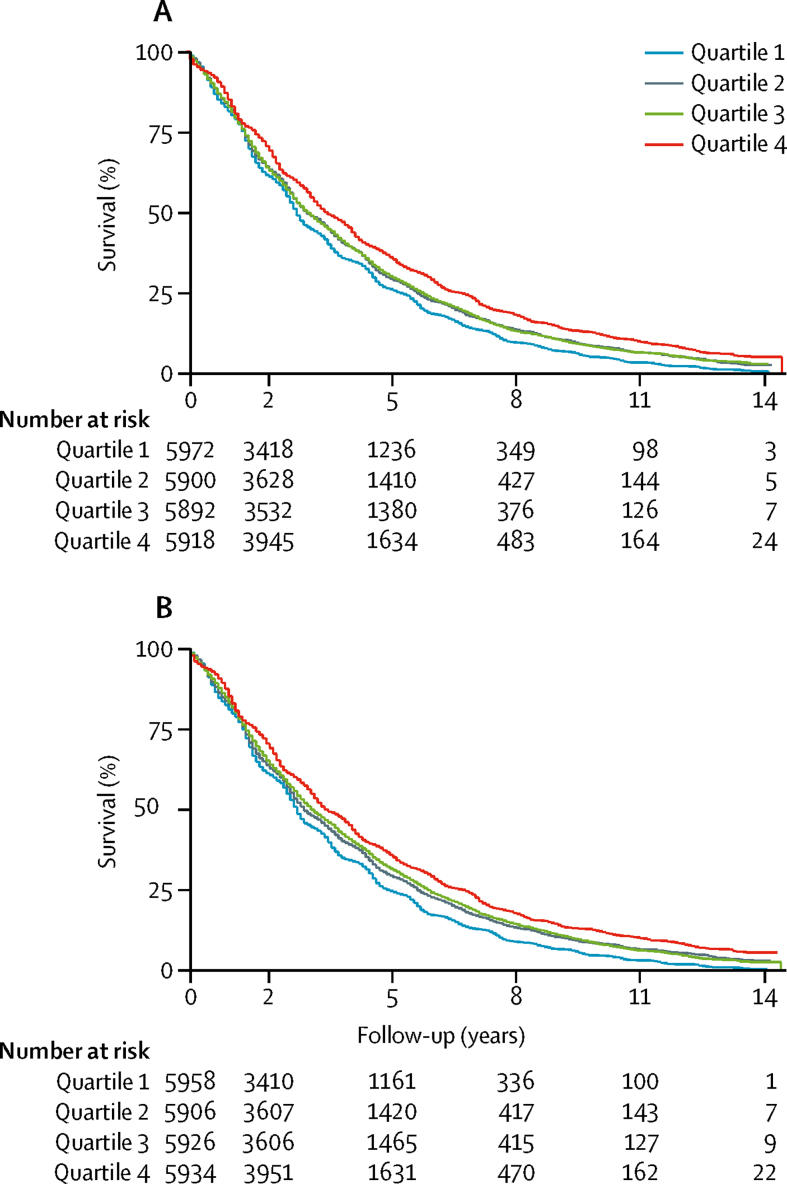
Kaplan-Meier survival curve for contemporaneous greenness exposure and all-cause mortality in the CLHLS, 2000–14 (n=23 754) (A) Survival curve for the 250 m radius. Log-rank p<0·0001. The median follow-up was 2·50 years (IQR 1·42–4·58) for quartile 1 (the lowest), 2·67 years (1·42–4·83) for quartile 2, 2·67 years (1·42–4·83) for quartile 3, and 2·92 years (1·67–5·25) for quartile 4 (the highest) of contemporaneous NDVI in the 250 m radius. (B) Survival curve for the 1250 m radius. Log-rank p<0·0001. The median follow-up was 2·50 years (1·33–4·50) for quartile 1 (the lowest), 2·67 years (1·42–4·83) for quartile 2, 2·67 years (1·42–4·92) for quartile 3, and 2·92 years (1·58–5·25) for quartile 4 (the highest) of contemporaneous NDVI in the 1250 m radius. CLHLS=China Longitudinal Healthy Longevity Survey. NVDI=Normalised Difference Vegetation Index.

Among 23 754 individuals, totalling 80 001 person-years of follow-up, we observed 18 948 deaths between June, 2000, and December, 2014. Figure 1 presents Kaplan-Meier survival curves by quartiles of contemporaneous NDVI and all-cause mortality, excluding accidental deaths. Individuals living in the highest quartile had longer survival than individuals in the lowest quartile. Additionally, individuals who were younger, female, of ethnic minority background, and married and living with their spouse had a higher adult socioeconomic status, and higher levels of social and leisure activity, and those who exercised tended to have a longer survival time than their counterparts (appendix).

**Figure 2:**
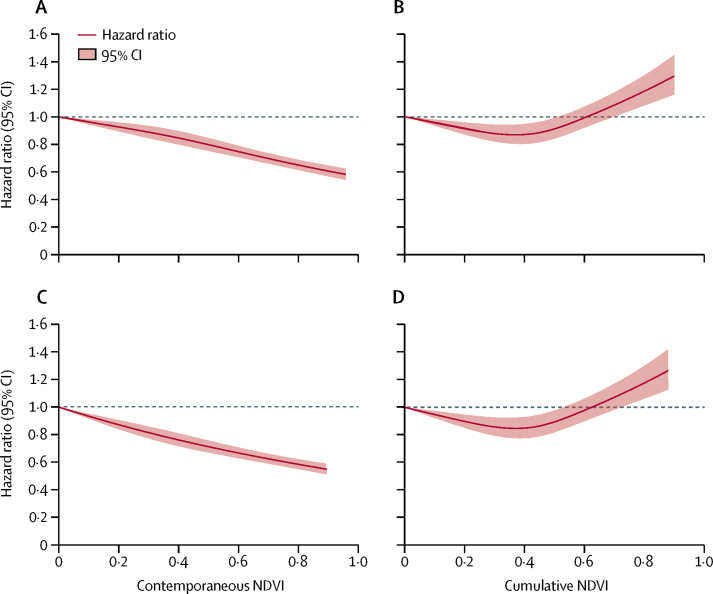
Cubic splines for greenness exposure and all-cause mortality in the CLHLS, 2000–14 (n=23 754, with 18 948 deaths)* (A) Cubic spline for contemporaneous NDVI in the 250 m radius. (B) Cubic spline for cumulative NDVI in the 250 m radius. (C) Cubic spline for contemporaneous NDVI in the 1250 m radius. (D) Cubic spline for cumulative NDVI in the 1250 m radius. CLHLS=China Longitudinal Healthy Longevity Survey. NVDI=Normalised Difference Vegetation Index. *286 participants with negative contemporaneous NDVI values and 87 with cumulative NDVI values were excluded in the analysis for the 250 m radius, as were 122 and two participants in the analysis for the 1250 m radius.

Table 2 presents HRs and 95% CIs for all-cause mortality in age-adjusted models, as well as in models fully adjusted for covariates. Individuals living in the highest quartile of contemporaneous NDVI for the 250 m radius had 27% lower mortality than those in the lowest quartile (HR 0·73, 95% CI 0·70–0·76; p<0·0001) in fully adjusted models. In the fully adjusted model, each 1-year increase in age was associated with a 6% increase in mortality (1·06, 1·05–1·06), suggesting that living in the highest quartile of greenness exposure compared with the lowest quartile has an equivalent effect on mortality as a 4·5-year reduction in age. These associations were consistent for the 1250 m radius (HR 0·70, 95% CI 0·67–0·74; p<0·0001). However, we observed no significant effects of cumulative NDVI when comparing the highest quartile with the lowest quartile in either the 250 m (HR 1·05 95% CI 1·01–1·10; p=0·236) or 1250 m radius categories (1·05, 1·00–1·10; p=0·171). Figure 2 shows cubic splines indicating linear association between contemporaneous NDVI and mortality but non-linearity between cumulative NDVI and mortality.

**Table 2:** Association of greenness exposure with all-cause mortality in the CLHLS, 2000–14 (n=23 754, with 18 948 deaths)

	**Contemporaneous greenness**			**Cumulative greenness**		
	NDVI median (range)	Age-adjusted HR (95% CI)	Fully adjusted HR (95% CI)	NDVI median (range)	Age-adjusted HR (95% CI)	Fully adjusted HR (95% CI)
**250 m buffer**						
Quartile 1	0·13 (−0·20 to 0·21)	1 (ref)	1 (ref)	0·19 (−0·15 to 0·29)	1 (ref)	1 (ref)
Quartile 2	0·29 (0·22 to 0·37)	0·92 (0·89 to 0·96)	0·90 (0·87 to 0·94)	0·39 (0·30 to 0·43)	1·06 (1·02 to 1·10)	1·04 (1·00 to 1·09)
Quartile 3	0·47 (0·38 to 0·56)	0·91 (0·87 to 0·94)	0·88 (0·84 to 0·92)	0·48 (0·44 to 0·51)	0·99 (0·95 to 1·03)	0·97 (0·93 to 1·02)
Quartile 4	0·68 (0·57 to 0·98)	0·76 (0·73 to 0·79)	0·73 (0·70 to 0·76)	0·57 (0·52 to 0·90)	1·05 (1·01 to 1·10)	1·05 (1·01 to 1·10)
p_trend_	..	<0·0001	<0·0001	..	0·102	0·236
**1250 m buffer**						
Quartile 1	0·15 (−0·09 to 0·22)	1 (ref)	1 (ref)	0·21 (−0·11 to 0·31)	1 (ref)	1 (ref)
Quartile 2	0·30 (0·23 to 0·37)	0·91 (0·87 to 0·94)	0·89 (0·85 to 0·92)	0·39 (0·32 to 0·43)	1·07 (1·02 to 1·11)	1·05 (1·01 to 1·11)
Quartile 3	0·46 (0·38 to 0·54)	0·87 (0·83 to 0·90)	0·83 (0·80 to 0·87)	0·48 (0·44 to 0·50)	1·01 (0·97 to 1·05)	1·00 (0·96 to 1·05)
Quartile 4	0·66 (0·55 to 0·91)	0·74 (0·71 to 0·77)	0·70 (0·67 to 0·74)	0·56 (0·51 to 0·88)	1·05 (1·01 to 1·09)	1·05 (1·00 to 1·10)
p_trend_	..	<0·0001	<0·0001	..	0·094	0·171

Data are median (range) or HR (95% CI). NDVI=Normalised Difference Vegetation Index. HR=hazard ratio.

Table 3 shows the association between mortality and changes in NDVI over time. 3174 (13·4%) participants lived in areas that became less green over the 14-year follow-up, and 5823 (24·5%) lived in areas that became greener over this time, whereas 14 757 (62·1%) had no significant changes in greenness. Among people living in regions with a significant increase in NDVI we found no effect of this increase on mortality for the 250 m radius or for the 1250 m radius (table 3) as compared with individuals living in regions with a decrease in NDVI. We found potential effect modification by sex (p_interaction_=0·015), financial status (p_interaction_=0·002), and physical activity (p_interaction_=0·005; appendix). Slightly higher protective effects of NDVI were seen in participants who were female, financially independent, and those who exercised than in those who were male, were not financially independent, or did not exercise (appendix).

**Table 3:** Changes in greenness exposure (NDVI) and all-cause mortality in the CLHLS, 2000–14 (n=23 754, with 18 948 deaths)

	**Participants**	**250 m buffer**				**1250 m buffer**			
		Age adjusted		Fully adjusted		Age adjusted		Fully adjusted	
		HR (95% CI)	p value	HR (95% CI)	p value	HR (95% CI)	p value	HR (95% CI)	p value
Significant decrease	3174 (13·36%)	1 (ref)	..	1 (ref)	..	1 (ref)	..	1 (ref)	..
Non-significant change	14 757 (62·12%)	0·99 (0·94– 1·03)	0·498	0·99 (0·95–1·04)	0·799	0·97 (0·93–1·01)	0·202	0·99 (0·94–1·03)	0·498
Significant increase	5823 (24·51%)	0·98 (0·94–1·03)	0·483	0·98 (0·94–1·03)	0·500	0·98 (0·93–1·03)	0·423	0·98 (0·93–1·03)	0·393

Data are n (%) or HR (95% CI). HR=hazard ratio. NDVI=Normalised Difference Vegetation Index.

## Discussion

In this prospective cohort study of the oldest-old population in China, we observed a protective association between contemporaneous greenness and mortality (27% decrease in the highest quartile compared with the lowest quartile). We found that the effect on mortality of living in the highest quartile compared with the lowest quartile of greenness has the equivalent magnitude of a 4·5-year reduction in age. We did not detect lower mortality among participants who lived in areas that increased in greenness compared with those living in areas that decreased in greenness over time. These findings carry substantial policy implications because China currently has one of the largest populations of older people in the world,^31^ and this demographic is expected to grow faster than in any other country.^32^ A previous study of NDVI measurements from 2000 to 2010 showed that China's greenness is increasing in inland regions, and decreasing in coastal areas, possibly due to rapid urbanisation and human factors.^33^ Moreover, higher cumulative average greenness appeared to be detrimental to mortality, although this association was not significant.

The finding of a protective association for contemporaneous greenness was stronger in this study than that shown in previous cohort studies, possibly because individuals in our study were much older and more prone to mortality events: the CLHLS study population, which is not a representative of the general age distribution in China, had a high percentage of centenarians. The Nurses' Health Study in the USA reported that women living in the highest quintile of contemporaneous greenness for a 250 m radius around their home had 12% lower mortality than did women living in the lowest quintile, at more than 8 years of follow-up.^12^ A national cohort study of Canadian adults showed an 8·5% reduction in mortality during 11 years of follow-up per IQR increase in NDVI within 250 m around their residential address.^13^ Another cohort study of urban residents in Ontario, Canada, reported a 5% reduction in all-cause mortality with a 4-year follow-up and a 500 m buffer.^14^ In Europe, the Swiss National Cohort of 4·2 million adults with 7·8 years of follow-up reported a 6% reduction in mortality with a 500 m NDVI, with a similar reduction for respiratory and cardiovascular disease mortality.^15^ In Asia, a cohort of senior citizens in Japan showed that available walkable green space was positively associated with longevity.^16^ A cohort of 3556 older adults aged 65 years and older in Hong Kong with 14 years of follow-up showed a 4% decrease in mortality per 10% increase in coverage of green space.^17^ However, this association was not found to be significant in another ecological study in Hong Kong.^34^ A systematic review of studies examining greenness and health suggested that increased greenness was consistently associated with decreased all-cause mortality.^34^ Our findings, in a much older population in China, are consistent with these other studies and indicate that contemporaneous greenness might be related to lower mortality.

Physical, mental, and immune-system factors could affect the association between greenness and mortality. In our population, individuals who were recorded at baseline as exercising had lower mortality over time than participants who did not exercise. Increased levels of greenness might provide opportunities for physical activity,^20^, ^35^, ^36^ which can help to reduce incidence of non-communicable diseases such as diabetes^37^ and cardiovascular diseases,^38^ leading to decreased mortality.^39^, ^40^ Some studies have shown that increased greenness exposure is related to reductions in major depressive disorders and improved mental health.^2^, ^35^, ^41^, ^42^ Exposure to greenness might promote social interaction and recovery from fatigue.^43^, ^44^ Additionally, greenness might contribute to better immune function through increased exposure to microorganisms associated with plants.^38^, ^45^, ^46^ However, our findings showed that cumulative average greenness was not significantly associated with mortality. Economic development, such as roadways and infrastructure (which are associated with reduced greenness) might possibly be related to improved access to health care, which is expected to lead to decreased mortality. Additionally, our results indicate that mortality in the oldest-old population might be more affected by acute rather than long-term exposures, because older people are more likely to spend more time immediately around their residence than young people.

Our study had a number of limitations. First, some individuals were lost to follow-up, although we believe they were unlikely to have changed residence because of their advanced age and the social benefits linked to the *hukou* (residency permit) household registration system.^20^, ^47^, ^48^ Furthermore, we excluded individuals who were lost to follow-up at the first survey because they did not contribute any time at risk to our analysis. In an analysis of baseline characteristics, we found that those who were lost to follow-up were more likely to be resident in or close to urban areas, which might have affected our results. Second, we did not have reliable data about cause-specific mortality in this cohort due to the self-report nature of the study design.^20^ We were not able to compare the effects of residential greenness on cause-specific mortality in our study with the findings from other studies. We relied on reports of death from family members rather than death certificates, which might have introduced recall bias in the mortality data in our study. This possibility of recall bias is a common concern in the longitudinal cohort studies, although CLHLS data have been validated as being reliable in previous studies.^20^, ^49^ Third, our cohort consisted of individuals who were already 80 years of age at the time of enrolment in our study so they were not representative of the general age distribution in China, and so their susceptibility to the natural environment might be different from that of the general population. Therefore, effect estimates of residential greenness might be different for that of other age groups. Fourth, because our exposure analysis was done via satellite, we did not have information about the specific types of vegetation participants were exposed to, nor did we have data about time activity patterns to know whether participants spent time in the green spaces. Therefore we could not assess which type of vegetation, if any, had greater benefits on mortality, and how exposure patterns influenced the association. Lastly, the interactive effects with those of climate change and indoor and ambient air pollution remain to be explored.^50^, ^51^

Our study had several strengths. First, to our knowledge, this study is the first to explore the association between greenness and mortality in the oldest-old population. Second, we believe this is the first study of its kind in China, and included participants from 22 provinces, covering the majority of regions in the country. Our study was a prospective cohort design, with a 14-year follow-up, and included a wide range of demographic and socioeconomic variables to control for potential confounding. Third, our study population is unique in that higher levels of greenness were associated with lower socioeconomic factors, and thus our findings are less likely to be explained by the confounding effects of socioeconomic status than those of other studies. Finally, our study of greenness might contribute to the assessment of China's burden of disease from environmental causes.^52^

In conclusion, in this prospective cohort study of the oldest old in China we observed that a higher contemporaneous exposure to greenness was associated with lower rates of non-accidental mortality. Similar protective effects were not observed for cumulative greenness exposure. These results indicate that greenness might be a protective factor for death in older people. As China undergoes urbanisation, our findings provide evidence to urban planners and health policy makers of the potential role of greenness in prolonging life.
