# Supplementary material for: Residential greenness and mortality in oldest-old women and men in China: a longitudinal cohort study
Source: Lancet Planet Health. 2019 Jan;3(1):e17–25. doi: 10.1016/S2542-5196(18)30264-X (PMC6358124; doi:10.1016/S2542-5196(18)30264-X)
Supplement: Supplementary appendix [file mmc1.pdf]

THE LANCET
Planetary Health

Supplementary appendix

This appendix formed part of the original submission and has been peer reviewed. We post it as supplied by the authors.

Supplement to: Ji JS, Zhu A, Bai C and others. Residential greenness and mortality in oldest-old women and men in China: a longitudinal cohort study. *Lancet Planet Health* 2019; **3:** e17–25.

Supplementary Table 1: Associated factors of survival status for CLHLS subjects: 2000-2014 (n=23 754, with 18 948 deaths)

**Predictors**	**HR (95%CI)**	**p value**
0.1*NDVI	0·95 (0·94, 0·95)	<0·001
Age	1·06 (1·05, 1·06)	<0·001
Female (Ref: males)	0·73 (0·71, 0·76)	<0·001
Han Chinese (Ref: others)	1·08 (1·01, 1·15)	0·021
Married and living with spouses (Ref: not married)	0·84 (0·80, 0·88)	<0·001
Childhood SES	1·01 (1·00, 1·03)	0·104
Adult SES	0·98 (0·96, 0·99)	0·012
Social and leisure activity	0·85 (0·84, 0·86)	<0·001
Smoking status (Ref: not smoke)	1·03 (0·99, 1·08)	0·177
Drinking status (Ref: not drink)	0·98 (0·94, 1·02)	0·299
Physical activity (Ref: not exercise)	0·90 (0·86, 0·93)	<0·001
Geographic region (Ref: Northeastern China)		
Central China	1·05 (0·98, 1·12)	0·193
Eastern China	1·02 (0·96, 1·09)	0·444
Northern China	1·06 (0·97, 1·16)	0·174
Northwestern China	1·05 (0·91, 1·21)	0·530
Southern China	0·97 (0·90, 1·03)	0·334
Southwestern China	1·01 (0·95, 1·09)	0·712

Supplementary Table 2: Hazard Ratios and 95% confidence intervals for contemporaneous NDVI (250m buffer) and cause-specific mortality in the CLHLS: 2000-2014 (n=23 754, with 18 948 deaths)**

Health outcomes	Greenness quartile 1	Greenness quartile 2	Greenness quartile 3	Greenness quartile 4	p for trend
Cancer (n=240)					
Cases	73	72	51	44	0·555
Fully-adjusted HR (95% CI)	Reference	0·67 (0·46, 0·97)	0·70 (0·46, 1·04)	1·16 (0·76, 1·75)	
Cardiovascular disease (n=1076)					
Cases	358	257	244	217	0·538
Fully-adjusted HR (95% CI)	Reference	1·01 (0·85, 1·20)	1·08 (0·91, 1·30)	1·04 (0·86, 1·25)	
Respiratory diseases (n=678)					
Cases	188	159	187	144	0·878
Fully-adjusted HR (95% CI)	Reference	1·02 (0·81, 1·27)	0·94 (0·75, 1·17)	1·05 (0·83, 1·33)	
Injuries and poisoning (n=74)					
Cases	21	24	16	13	0·997
Fully adjusted HR (95% CI)	Reference	0·41 (0·18, 0·92)	0·48 (0·20, 1·12)	0·92 (0·38, 2·24)	
Endocrine, nutritional, metabolic and immune diseases (n=265)					
Cases	63	74	71	57	
Fully adjusted HR (95% CI)	Reference	0·93 (0·64, 1·37)	0·88 (0·61, 1·27)	0·89 (0·60, 1·32)	0·517
Urinary and reproductive diseases (n=62)					
Cases	15	14	15	18	
Fully adjusted HR (95% CI)	Reference	0·63 (0·24, 1·69)	1·22 (0·42, 3·52)	1·23 (0·52, 2·92)	0·497
Infectious diseases (n=55)					
Cases	9	15	15	16	0·616
Fully adjusted HR (95% CI)	Reference	1·01 (0·28, 3·67)	1·28 (0·38, 4·38)	1·20 (0·37, 3·92)	

*Note: There are few deaths caused by dementia (n=13) and neurosis (n=13). The sample size is not big enough for the Cox proportional hazard models.

** Cause-specific mortality in the CLHLS was higher underestimated, underreported, and only available before 2005 wave. Therefore, the sum of specific causes of death in this table is fewer than total number of death.
